# Enhancing Deep Learning Model Explainability in Brain Tumor Datasets Using Post-Heuristic Approaches

**DOI:** 10.3390/jimaging10090232

**Published:** 2024-09-18

**Authors:** Konstantinos Pasvantis, Eftychios Protopapadakis

**Affiliations:** Department of Applied Informatics, University of Macedonia, Egnatia 156, 546 36 Thessaloniki, Greece

**Keywords:** trustworthiness, explainability, brain tumor detection

## Abstract

The application of deep learning models in medical diagnosis has showcased considerable efficacy in recent years. Nevertheless, a notable limitation involves the inherent lack of explainability during decision-making processes. This study addresses such a constraint by enhancing the interpretability robustness. The primary focus is directed towards refining the explanations generated by the LIME Library and LIME image explainer. This is achieved through post-processing mechanisms based on scenario-specific rules. Multiple experiments have been conducted using publicly accessible datasets related to brain tumor detection. Our proposed post-heuristic approach demonstrates significant advancements, yielding more robust and concrete results in the context of medical diagnosis.

## 1. Introduction

Deep learning models’ capacity to model complicated patterns on various fields, such as medical imaging, has demonstrated great potential for prognostic and diagnostic purposes [1,2,3,4,5,6]. Deep learning’s extensive capabilities have made progress in medical image analysis possible, allowing for the more accurate and efficient diagnosis of a variety of illnesses.

However, the deployment of deep learning models in medical image analysis is not without challenges [7,8]. One major issue with these models is their lack of explainability. Because deep neural networks are complex, their decision-making processes are not frequently transparent, which makes it difficult for medical experts to understand and accept the outcomes. This problem is particularly important for medical applications, because misinterpretation might have serious consequences.

Researchers have looked into a number of ways to improve the interpretability of deep learning models in order to close the explainability gap, especially when it comes to medical imagery [9,10,11]. Such approaches could include (a) Model-specific methods, e.g., saliency maps or activation maximization [12,13], or (b) Model-agnostic methods, e.g., partial dependence plots or surrogate models [14,15,16,17]. Even though progress has been achieved in this area, there is always opportunity for enhancement, particularly when it comes to improving the results’ interpretability for certain applications.

This paper explores the relationship between medical image analysis and deep learning, specifically focusing on enhancing the explainability of classification models for detecting brain tumors in MRI images. Understanding the difficulties in obtaining results that are transparent, we use an explainability method that is specific to the complexities of medical image analysis. Most importantly, we contribute to a later improvement of this method with the goal of offering more accurate and useful information on the existence of brain tumors.

## 2. Related Work

Various CNN architectures, such as AlexNet [18] and VGGNet [19], have been influential in classifying medical images into distinct categories. Transfer learning methods have also made it easier to apply previously learned CNN models to medical image classification tasks, which has enhanced diagnostic capabilities. AlexNet, for example, has shown success in classifying skin lesions in dermatological images, contributing to the early identification of skin cancers [20].

The application field may vary, from suspicious and abnormal ardiotocographic recordings [21] to glaucoma detection [22]. The former case is important for monitoring the health of both the mother and the fetus during pregnancy. The proposed method improved time complexity, a crucial factor in clinical settings, by combining AlexNet with support vector machines (SVMs) at the fully connected layers. The latter case involved the development of image analysis diagnostic tools.

Another notable example of transfer learning regarding classification is the adaptation of a pre-trained ResNet model for the detecting COVID-19 in chest X-ray images [23]. The model was evaluated using both binary classification and multi-class classification methods, and all of the results indicate that the use of the model can assist medical experts in precisely detecting COVID-19 cases.

In the field of neuroscience, in order to mitigate the serious health risks that brain tumors offer, fast and accurate brain tumor detection is essential. Early detection has a major impact on treatment outcomes, in addition to allowing timely intervention. In this context, deep learning approaches have transformed the processing of brain tumor images. In particular, architectures like U-Net have proven useful in accurately identifying brain tumors from MRI scans, supplying vital data for treatment planning [24,25]. Moreover, the categorization of brain images has been improved with the use of custom CNN architectures and transfer learning methods.

For example, a study utilized transfer learning, using the advantages of existing pretrained models, in order to accurately classify brain tumors from MRI images [26]. In addition, there are multiple research projects that have focused on utilizing transfer learning methods in order to correctly identify brain tumors from MRI scans, resulting in more correct predictions than the traditional use of CNNs [27,28,29].

As deep learning techniques are still transforming medical image processing, there is an increasing need for these complicated models’ decisions to be transparent and understandable. Explainability, also known as the interpretability of artificial intelligence (AI) systems, is becoming more and more important, especially in healthcare applications where it is critical to comprehend the reasoning behind a diagnosis [30,31]. Explainable AI (XAI) aims to provide information about how and why specific conclusions are made from complicated datasets, simplifying the decision-making processes of deep learning models in the larger context of image processing.

CNN’s use of attention mechanisms is a popular example of explainability in AI. Attention mechanisms give models the ability to concentrate on particular areas of an image, offering a type of interpretability by highlighting the input elements that the model considers most important to its judgment. There are many algorithms that utilize attention mechanisms in a medical imaging context, and there is a comperative study among them examining their results [32].

Additionally, saliency maps are frequently used to show which areas of a picture contribute most to the output of the model. Saliency-based methods are much more often used in medical imaging, given the fact that the practitioner can understand the reasoning behind the prediction for every image.

Regarding brain tumor detection from images, there are many saliency-based methods used for explainability, including gradient-based or perturbation-based [33]. Local Interpretable Model-agnostic Explanations (LIMEs) [34] is a perturbation-based method that finds the segments contributing to a model’s prediction for a given image. There are already research projects that have focused on explaining decisions made from pre-trained deep learning models [35,36] using this method, but this method alone may not produce a proper explanation to make things better for the user.

### 2.1. Research Challenges

Using the LIMEs library for medical image interpretation poses several significant challenges. A primary obstacle is creating segments that correspond well with the actual content of the image [37]. In medical imaging, where precise details hold crucial diagnostic information, segments that fail to capture relevant features may erroneously appear as significant as those containing critical information. This mismatch can lead to confusion among users attempting to gain insights from the model’s predictions, compromising the trustworthiness of the explanations provided.

Furthermore, LIMEs is highly sensitive to even minor changes in the input image. The stability of LIMEs’ explanations is highly susceptible to issues such as the introduction of noise, which can cause significant changes in the explanations. This instability introduces uncertainty into the interpretation process, potentially leading to inconsistencies in outcomes and reducing the trustworthiness of LIMEs’ interpretations, especially in the context of medical image analysis. As a result, establishing the reliability and consistency of LIMEs’ explanations is critical for building trust in its ability to facilitate accurate and reliable diagnoses in medical imaging applications.

In addition to the challenges associated with the LIME library, the integration of post-refinement mechanisms introduces its own set of complexities. Another notable challenge is the selection and optimization of appropriate post-refinement techniques adapted to the specific properties of medical images. Given the diverse nature of medical imaging data, which ranges from changes in imaging modalities to variations in anatomical structures and diseases, establishing efficient refining methods that transfer well across different datasets poses a substantial challenge.

Furthermore, the computational strain associated with post-refinement procedures should be carefully considered, especially in real-time clinical contexts where rapid diagnostic choices are critical. Balancing the need for enhanced interpretability with the computing efficiency required for practical deployment is still a major challenge in the development of post-refinement processes for medical image analysis.

### 2.2. Our Contribution

Building on the existing literature, our study addresses a critical gap by introducing a novel end-to-end architecture specifically designed to enhance the explainability of deep learning models using image post-processing techniques. Unlike current frameworks, our approach systematically improves the interpretability of image-based explanations through customized mechanisms. This research provides a reliable solution by integrating a refined post-processing step into the explainability pipeline.

Our primary contribution is the creation of a robust refinement approach designed to address the challenges inherent in existing frameworks, particularly in the context of medical image analysis. By incorporating this refinement step, we want to improve the interpretability and reliability of the explanations given by machine learning models, allowing for more informed decision making in clinical contexts.

Furthermore, our findings emphasize the relevance of end-to-end solutions in deep learning explainability, highlighting the need for the efficient integration of post-processing approaches to improve model interpretation consistency. Through our proposed architecture, we seek to establish a standardized approach for refining explainability results, ultimately advancing the transparency and trustworthiness of deep learning models in medical image analysis and beyond.

## 3. Proposed Methodology

Let $I\in Z^{w\times h}$ be a grayscale image, originating from an MRI scanner, and t∈{0,1} is the decision of a deep learning model (Section 3.1) regarding the existence or not of a tumor. Then, a model-agnostic technique (Section 3.2) generates a heatmap, $H\in R^{w\times h}$, over image *I*. The heatmap indicates regions of interest, over *I*, which have contributed to generating the output *t*. Ideally, the most prominent regions should include a high portion of the tumor area, giving the physician a proper explanation.

**Figure 1:**
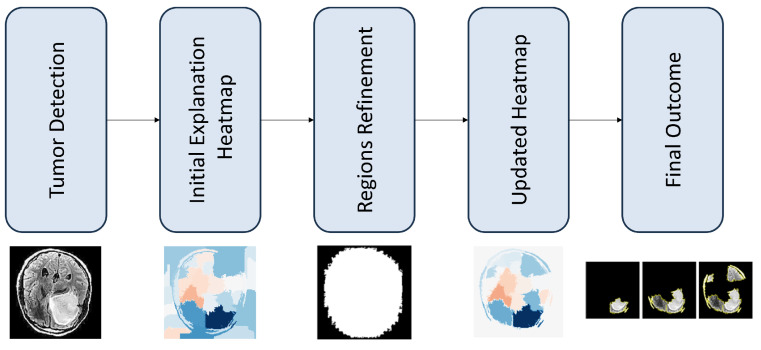
Proposed methodology.

The adopted approach introduces an additional refinement mechanism (Section 3.4), R(I,H), which considers both *I* and *H*, as well as eliminates non-informative segments of *H*, based on a combination of image morphology operations and post-processing heuristics so that $R\left(I,H\right)\to H^{\left(R\right)}$. $H^{\left(R\right)}$ is the refined version of *H*, retaining the most appropriate segments related to brain and tumor geometry after using the techniques explained (Section 3.3). Figure 1 demonstrates the process.

### 3.1. Employed Deep Learning Architectures

In this work, we handle the brain tumor detection as a binary classification problem using as input a grayscale image, say *I*. We try to establish a prediction model, f(x)→t, t∈{0,1}, so that given the image *I*,

$$f\left(I\right)=\left\{\begin{array}{cc} 0, & \mathrm{if}\;I\;\mathrm{has}\;\mathrm{not}\;\mathrm{brain}\;\mathrm{tumor}\\ 1, & \mathrm{if}\;I\;\mathrm{has}\;\mathrm{brain}\;\mathrm{tumor} \end{array}\right.$$

The process incorporates the paradigm of transfer learning, since it can provide significant advantages in medical applications [38]. In particular, three deep learning pre-trained models were used: InceptionV3 [39], ResNet50V2 [40], and NasNetLarge [41].

InceptionV3 is a CNN architecture designed for efficient and accurate image classification tasks. Its modules include parallel convolutional operations, allowing the network to capture features at various scales. The architecture is characterized by the use of global average pooling, replacing fully connected layers, which aids in reducing the model’s parameter count. InceptionV3 employs rectified linear unit (ReLU) activation functions to introduce non-linearity. Batch normalization is also integrated, contributing to faster convergence during training.

ResNet50V2, part of the ResNet (Residual Network) architecture family is a deep convolutional neural network created to help with the challenges of training very deep networks. By introducing skip or residual connections, ResNet enables the flow of information directly from one non-adjacent layer to another.

NasNetLarge is a neural network architecture designed through automated architecture search methods. As opposed to manually designed architectures, NasNet is produced by utilizing techniques from reinforcement learning to explore a large search space of possible architectures. It utilizes a combination of normal and reduction cells that are repeatedly stacked to form the overall network structure. Complex patterns and representations in images are successfully captured by the architecture thanks to skip connections and effective utilization of computational resources.

In our study, we used the same parameters for each of these pre-trained models. The pre-trained layers of each model were frozen to retain their weights from the Imagenet Dataset, and a custom head was added in order to adapt to our dataset. This means that, after the first output of each pre-trained model, we pass this in new layers that combine global average pooling and desnse layers concluding with a softmax layer. The training involved utilizing the adam optimizer, as well as categorical crossentropy loss, over 10 epochs. An early stopping criterion was implemented in order to reduce overfitting.

### 3.2. Model’s Explainability

The Lime Image Explainer (LIE) is employed for creating an explanation, given an image $I_{c}$, which has been classified as positive to cancer, i.e., $t_{c}=1$. At first, given the image $I_{c}$, the LIE creates a new set of images, say $Z=\{I_{c}^{\left(1\right)},...,I_{c}^{\left(n\right)}\}$, with the same dimensions w×h. The process can be summarized as follows:

(a) A segmentation algorithm, e.g., QuickShift, Slic, or Felzenszwalb, operates over image $I_{c}$, generating *d* segmented areas. This number denotes the number of segments produced by the LIE using one of the above segmentation algorithms, and it can vary from image to image.
(b) Then, a new image instance, $I_{c}^{\left(k\right)}$, is created by maintaining a random number, m<d, of the original segments, and once again, it depends on the number m and on the image that is used at the moment.
(c) Repeat the process until a predefined number of images are generated. This number is taken as a parameter into the LIE. In this study, we used 1000 new generated images.

The proposed approach generates multiple copies of the image $I_{c}$ with missing areas, corresponding to some of the *d* segments. Theoretically, such a process may generate up to n=d0+d1+⋯+dd new image instances.

Then, we use the prediction model, described in Section 3.1 to predict the outcomes using *Z* as inputs. Each instance $I_{c}^{\left(k\right)}\in Z$ is fed to the deep learning model and the corresponding output such that $t^{\left(k\right)}$ is generated. The LIE calculates the weights corresponding to each segment area, creating a sparse linear model, which approximates the outputs of the deep learning model f(x) [42]. That way, the weights highlight the importance of each segment in contributing to the model’s decision, providing a local and interpretable understanding of the black box model’s behavior within a specific region of the input space.

**Figure 2:**
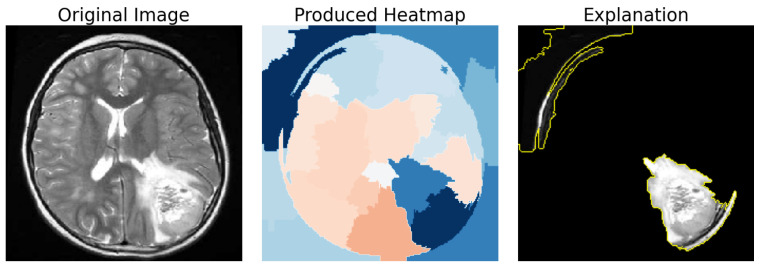
Demonstrating the overlap of the 3 most important segments (**right**) given an input image (**left**) and the LIE heatmap (**center**).

The result is a heatmap is of the form $H=\{S_{i}:Importance_{i},\cdots ,S_{j}:Importance_{j}\}$, where $S_{i}$ represents the ID of the segment, and $Importance_{i}$ denotes the corresponding importance value or the weight assigned to that segment by the LIE. Our interest is in identifying the best *n* areas from the heatmap *H* provided by the explanation. The number *n* is selected based on various performance metrics, which are explained in Section 4.2. Figure 2 illustrates the above using a single image as an example, with the best three segments obtained from the heatmap that was produced.

### 3.3. Brain Area Segmentation

A quick examination of the generated heatmaps, compared to the input images, demonstrates a significant flaw in terms of explainability. It appears that certain segments were outside the bounds of the brain, having no meaningful contribution, from a medical expert perspective, in the generated explanation. As such, a mitigation strategy could be considered. In this study, multiple image operators, i.e., filters, have been considered to improve and refine the explanations’ interpretability.

The core idea lies in the successful segmentation of the brain area, given an image *I*, using image processing techniques. In this case, the problem at hand could be addressed as an edge detection problem. There are multiple explicit approaches for the identification of edges in an image like Laplace (Section 3.3.1), Sobel (Section 3.3.2), and Canny (Section 3.3.3), or there are implicit ones like thresholding Otsu and Li (Section 3.3.4).

Subsequently, based on the edges provided from the previous algorithms, a new binary brain mask is created. The mask is generated by identifying and extracting the largest contour from the detected edges. This mask provides a rough approximation of the brain area, and it matches the size of the original image. The resulting brain mask, denoted as BrM, serves as a crucial element in our refinement process, providing a clear binary definition of the brain’s spatial extent.

#### 3.3.1. Laplacian Filter

The Laplace filter is one of the many methods used for edge detection.The operator relies on the second derivative to highlight sudden changes in intensity, helping identify important features in the image. The formula for the Laplace filter in image processing is as follows:

$$\begin{array}{cc} \nabla^{2}I\left(x,y\right) & =\sum_{i=-1}^{1}\sum_{j=-1}^{1}w\left(i,j\right)\cdot I\left(x+i,y+j\right)\; \end{array}$$

where $\nabla^{2}I\left(x,y\right)$ is the intensity of the output pixel (x,y) after applying the Laplace filter, I(x+i,y+j) represents the intensity of the input pixel at coordinates (x+i,y+j), and w(i,j) are the weights of the Laplacian filter mask. Typically, the weights are defined as follows:

$$w_{i,j}=\left[\begin{array}{ccc} 0 & 1 & 0\\ 1 & -4 & 1\\ 0 & 1 & 0 \end{array}\right]$$

**Figure 3:**
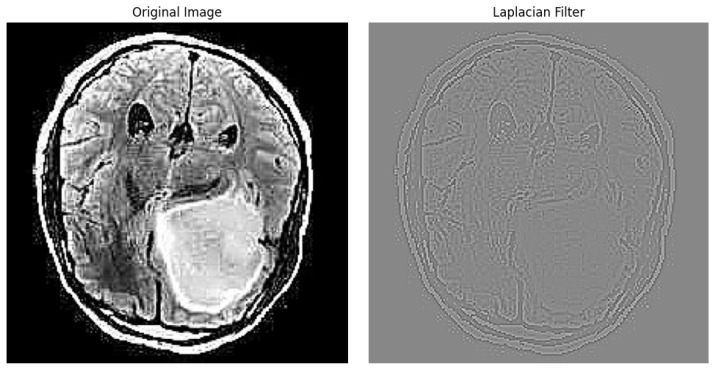
Edge Detection example using Laplacian Filter.

The result of an image after applying the Laplacian filter can be seen in Figure 3.

#### 3.3.2. Sobel

The sobel operator is another commonly used technique, for edge detection, that calculates an approximation of the gradient of the image intensity function. This technique has two kernels: the horizontal $G_{x}$ and the vertical $G_{y}$. Typically, the two kernels are as follows:

$$\begin{array}{c} \begin{array}{cccc} G_{x} & =\left[\begin{array}{ccc} -1 & 0 & 1\\ -2 & 0 & 2\\ -1 & 0 & 1 \end{array}\right] & ,\;G_{y} & =\left[\begin{array}{ccc} -1 & -2 & -1\\ 0 & 0 & 0\\ 1 & 2 & 1 \end{array}\right] \end{array} \end{array}$$

The intensities of each pixel, for  both the horizontal $I_{hor}\left(x,y\right)$ and vertical direction $I_{ver}\left(x,y\right)$, are defined using the same formula as the Laplace operator:

$$\begin{array}{c} \begin{array}{cccc} I_{hor}\left(x,y\right) & =\sum_{i=-1}^{1}\sum_{j=-1}^{1}G_{x}\left(i,j\right)\cdot I\left(x+i,y+j\right), & I_{ver}\left(x,y\right) & =\sum_{i=-1}^{1}\sum_{j=-1}^{1}G_{y}\left(i,j\right)\cdot I\left(x+i,y+j\right) \end{array} \end{array}$$

**Figure 4:**
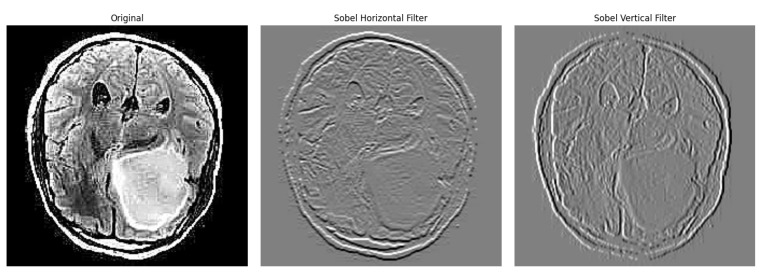
Edge detection example using sobel filters.

Finally, the gradient magnitude M(x,y) for each pixel is computed using the intensities of the image M(x,y), where $M\left(x,y\right)=\sqrt{{\left(I_{hor}\left(x,y\right)\right)}^{2}+{\left(I_{ver}\left(x,y\right)\right)}^{2}}$, and thresholding is then applied to the gradient magnitude in order to highlight edges. An example of vertical and horizontal sobel filters can be seen below in Figure 4.

#### 3.3.3. Canny Edges

The canny edge detector uses a Gaussian filter to smooth the image, and then it typically uses the sobel operator in order to define the gradient magnitude and gradient direction at each pixel. The direction θ at each pixel is calculated using the $I_{hor}$ and $I_{ver}$ using the following formula:

$$\theta \left(x,y\right)=arctan(I_{ver}\left(x,y\right),I_{hor}\left(x,y\right),$$

where arctan is the arctangent function that returns the angle between the intensity of the pixel in the x axis and the intensity of the pixel in the y axis.

**Figure 5:**
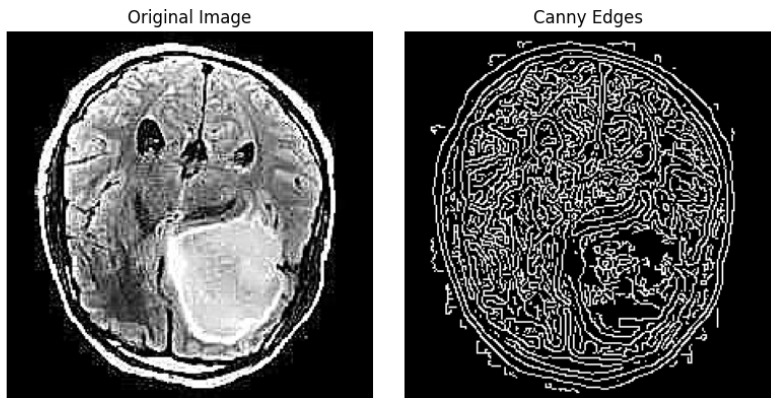
Edge detection example using canny filter.

After this step, this technique involves thinning the edges to a single pixel width, iterating over all pixels in the gradient magnitude image and suppressing the gradient values of all pixels except the local maxima in the direction of the gradient. The result of this technique is similar, as shown in Figure 5.

#### 3.3.4. Li’s and Otsu’s Thresholding

A key method in image processing is thresholding, which is used to distinguish objects or areas of interest from the background according to pixel intensity levels. It works by turning color or grayscale images into binary images, in which pixels are categorized as background or foreground (object of interest) based on whether or not they satisfy thresholds or intensity requirements.

The process of thresholding involves setting a threshold value, depending on the intensity of image’s pixels, which acts as a dividing line between the foreground and background pixels. Pixels with intensity values above the threshold are assigned to the foreground, while those below the threshold are assigned to the background. This results in a binary image, where foreground pixels are typically represented as white (or 1) and background pixels as black (or 0).

Mathematically speaking, for a given image *I*, a threshold thr based on an algorithm *A* is found, and the result is a new image $I^{\prime }$ with pixel values $I^{\prime }\left(x,y\right)$ such that

$$I^{\prime }\left(x,y\right)=\left\{\begin{array}{cc} 0, & \mathrm{if}\;I(x,y)<thr\\ 1, & \mathrm{if}\;I(x,y)\geq thr \end{array}\right.$$

In this study, the algorithms used for thresholding were Li’s and Otsu’s thresholding.

Both algorithms use the same principles. Otsu’s thresholding aims to maximize the variance between the foreground *F* and background *B*. That is, for a given threshold thr, the algorithm calculates the variance between the two classes as follows:

$$V\left(t\right)=p_{F}\left(thr\right)\cdot p_{B}\left(thr\right)\cdot {\left[\mu_{B}\left(thr\right)-\mu_{F}\left(thr\right)\right]}^{2},$$

where

- $p_{F}\left(thr\right)$ and $p_{B}\left(thr\right)$ are the percentage of foreground and background pixels for the given threshold thr;
- $\mu_{B}\left(thr\right)$ and $\mu_{F}\left(thr\right)$ are the mean of the background and foreground pixels intensity for the given threshold thr,

The threshold thr that maximizes the variance is selected, and the result is a binary image, as explained before.

Li’s thresholding, aims to minimize the cross-entropy between the intensity values of the foreground pixels and the mean intensity value of the foreground, as well as between the intensity values of the background pixels and the mean intensity value of the background. This essentially means that the threshold should be chosen in such a way that the difference between the intensity values of pixels within a region (foreground or background) and the mean intensity value of that region is minimized.

Mathematically speaking, the algorithm tries to find the optimal threshold thr using the following:

$$argmin_{t}\left(H_{F}\left(thr\right)+H_{B}\left(thr\right)\right),$$

where the following values are defined:

- $H_{F}\left(thr\right)$ is the cross-entropy between the foreground region and the foreground mean intensity using threshold thr;
- $H_{B}\left(thr\right)$ is the cross-entropy between the background region and the background mean intensity using threshold thr.

**Figure 6:**
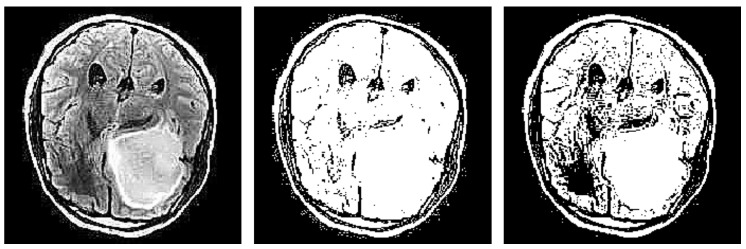
Generated binary masks using Li’s and Otsu’s thresholding.

We can take a glimpse at the produced results for a specific image after using these two techniques in Figure 6.

### 3.4. Post-Processing Refinement Mechanisms

After the production of the brain mask for an image, our refinement mechanism introduces a criterion for retaining segments produced by LimeImageExplainer for the same image. The LimeImageExplainer produced a heatmap *H* (Section 3.2). We then only retained the segments according to the following formula:

$$SelectedSegments=\{S_{i}|Pixels\left(S_{i}\cap BrM\right)\geq 0.8\cdot Pixels\left(S_{i}\right)\}$$

The result is a new heatmap $H^{\left(R\right)}$, which is defined by a new dictionary that is as follows:

$$H^{\left(R\right)}=\left\{S_{i}:Importance_{i}^{\prime },\cdots ,S_{j}:Importance_{j}^{\prime }\right\},$$

where this time, the $Importance_{i}^{\prime }$ is the same with the $Importance_{i}$ if $Segment_{i}$ was retained, and it is 0 otherwise. This refined heatmap $H^{\prime }$ provides a more accurate representation of the segments contributing to the model’s prediction.

Algorithm 1 summarizes the refinement process. Practically speaking, the refinement process operates over a LIMEs heatmap and recalculates the segments’ importance. Any heatmap segment that does not have a high overlap with the brain mask, produced as explained in Section 3.3, is considered non-informative, and its importance is set to 0. In this study, a threshold overlap value of 80% was selected. If a segment meets this criterion, i.e., 80% of its pixels are brain pixels, it retains its original importance, reflecting its relevance to the model’s prediction.
**Algorithm 1** Post-processing refinement mechanism. 1:**Input:** *H* (LIMEs-generated heatmap), BrM (Brain Mask) 2:**Output:** $H^{\left(R\right)}$ (Refined heatmap) 3:**for** each segment $S_{i}$ in *H* **do** 4:    $overlap\leftarrow \mathrm{Pixels}(S_{i}\cap BrM)$ 5:    $threshold\leftarrow 0.8\cdot \mathrm{Pixels}(S_{i})$ 6:    **if** overlap≥threshold **then** 7:          $Importance_{i}^{\prime }\leftarrow Importance_{i}$ 8:      **else** 9:          $Importance_{i}^{\prime }\leftarrow 0$10:  **end if**11:**end for**12:$H^{\left(R\right)}\leftarrow \left\{S_{i}:Importance_{i}^{\prime }\;\mathrm{for}\;\mathrm{each}\;\mathrm{segment}\;S_{i}\right\}$13:**return** $H^{\left(R\right)}$

We conducted a small test with approximately 50 images ranging from 50% to 90% in order to investigate the possible effects that these percentages may have. We found out that when we used a small percentage, the segments that remained were quite uninformative for the algorithm, because there were many segments that were outside of the brain area. Also, when we used a high percentage, the segments that were retained were mostly inside of the brain region, but we were losing many segments that had a small part of them outside of the brain. As such, the 80% value was selected. This method effectively filters out extraneous information, ensuring that only the most pertinent segments contribute to the final explanation. As a result, the refined heatmap provides a clearer and more focused representation of the segments that truly influence the model’s decision-making process.

## 4. Experimental Setup

The proposed scheme was evaluated using a publicly available dataset, which is described in the end of this article. The objective of the methodology was to refine the LIE-generated heatmaps by removing topology-related inconsistencies. All of the experiments conducted were employed using Python utilizing public libraries (Tensorflow, Skimage, Sklearn, Shapely, Lime, and Matplotlib). The computations were performed in Google Colab with GPU acceleration enabled.

At this point, we need to stress that the setup was arranged to provide robust conclusions about the impact of combining different image segmentation algorithms and edge detection techniques within the LIMEs framework. Our approach aimed to refine and enhance the interpretability of model predictions. The optimal configuration of these methodologies will be explored in future work to further improve the reliability and accuracy of tumor detection.

The following sections present the datasets employed in this study and the approach followed to form training and evaluation sets. Then, we present the details of selecting the classification model that performed best in the specific dataset. Finally, we discuss the obtained results and their implications for improving the interpretability of the deep learning models examined in our study.

### 4.1. Dataset Pre-Processing

Prior to training the models, the dataset underwent pre-processing steps to ensure consistency and suitability. The images were resized to 224 × 224 pixels and normalized to standardize the pixel values within the range of 0 to 1. To improve the dataset’s quality and diversity, duplicate images were removed, resulting to a total of 4015 images. Furthermore, a Stratified K-Fold validation strategy with five splits was used. This methodology ensures a robust evaluation of the deep learning models’ performance by guaranteeing that each fold retains the same distribution of classes as the original dataset.

### 4.2. Performance Metrics

Each deep learning model’s performance was evaluated using typical metrics like accuracy, precision, recall, and F1 score. These metrics rely on key elements such as True Positive (TP) values, True Negative (TN) values, False Positive (FP) values, and False Negative (FN) values. These metrics are defined as follows:

$$Accuracy=\frac{TP+TN}{TP+TN+FP+FN}$$

$$Precision=\frac{TP}{TP+FP}$$

$$Recall=\frac{TP}{TP+FN}$$

$$F1Score=2\cdot \frac{Precision\cdot Recall}{Precision+Recall}$$

In addition to these metrics, we introduced a new metric to evaluate how well the segments from LimeImageExplainer matched the presence of tumors. Using the VGG Image Annotator, manual annotations of the tumor were performed on 271 images out of the 471 infected instances in the test set. These images were selected for their relative ease of annotation. This custom metric aims to quantify the percentage of the brain tumor included in the explanation’s segments, and we will refer to it as “Tumor Segment Coverage”.

**Figure 7:**
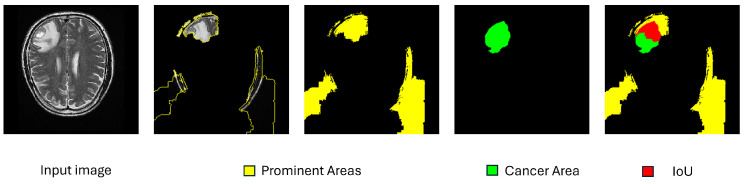
An example of tumor coverage calculation.

To be more specific, after the use of the VGG Image Annotator, a new mask that represents the location of the tumor was created. This means that a pixel of the original image (x,y) belongs in the Tumor Mask if and only if this pixel is inside of the tumor polygon that is created. With this mask, to find the Tumor Segment Coverage (TSC), we calculated the percentage of pixels that were both part of the mask and the tumor annotation. Figure 7 demonstrates such a case with a Tumor Segment Coverage of 51.75%.

**Figure 8:**
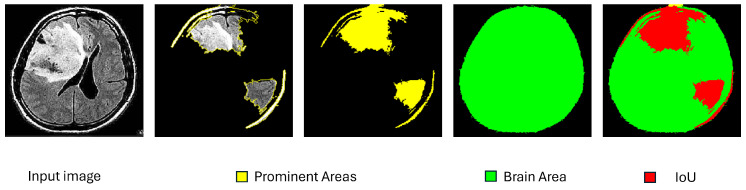
An example of brain coverage calculation.

In addition we used another metric called “Brain Segment Coverage” (BSC), and it defines the percentage of brain mask covered by the explanation’s segments. This means that, after we produced the refined explanation, we calculated the percentage of the pixels that were part of the explanation and also belonged in the brain mask that was created using an edge detector, as explained in Section 3.4. Figure 8 demonstrates such a case with a brain coverage score of 21.92%.

### 4.3. Segmentation-Based Refinement Impact

This section demonstrates the possible impact, for the proposed refinment methodology, as discused in Section 3.4. In particular, we started by selecting the best classification model and then produced explanations for the MRI images using the LIE. We then examined the results produced after the use of our refinement method.

**Figure 9:**
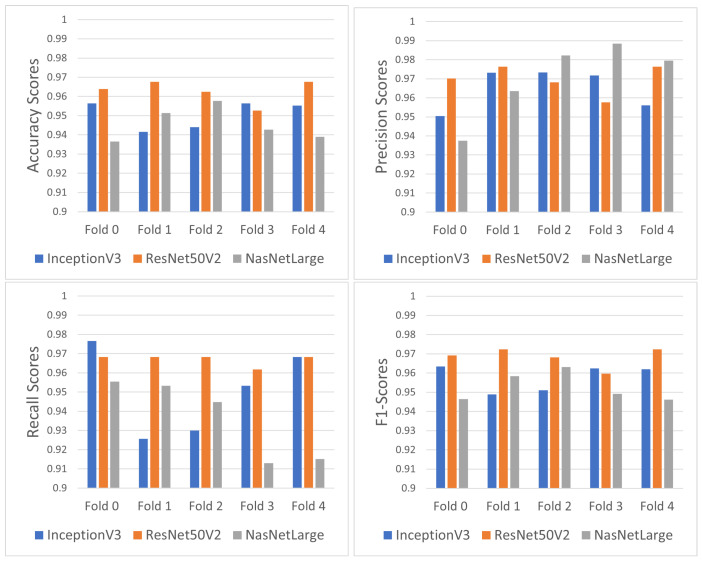
Classification performance scores for the utilized approaches.

Table 1 and Figure 9 demonstrate the performance scores for the models’ predictions for every fold. ResNet50V2 appears to be the most prominent classifier in terms of its F1 score.

To compare the pre-trained models, we conducted a statistical Mann–Whitney test on their F1 scores, considering both the scores obtained during cross-validation and after. The test showed that ResNet50v2 outperformed both InceptionV3 (*p* = 0.02) and NasNetLarge (*p* = 0.008). However, there was no significant difference between InceptionV3 and NasNetLarge (*p* = 0.39). Given these results, we chose ResNet50v2 for further analysis in the explainability part.

As mentioned before, the LimeImageExplainer was employed to provide explanations for model predictions before and after the proposed refinement. We used three segmentation algorithms in order to separate the image into segments: QuickShift, Slic, and Felzenszwalb. We also used five edge detection techniques, as mentioned in Section 3.3. There were two quantitative criterion used for the performance of the adopted approaches: (a) BSC and (b) TSC. The former validates whether the applied image processing techniques correctly identify the brain areas. The latter demonstrates how practical is, for the medical experts, the *n* most important image segments.

**Figure 10:**
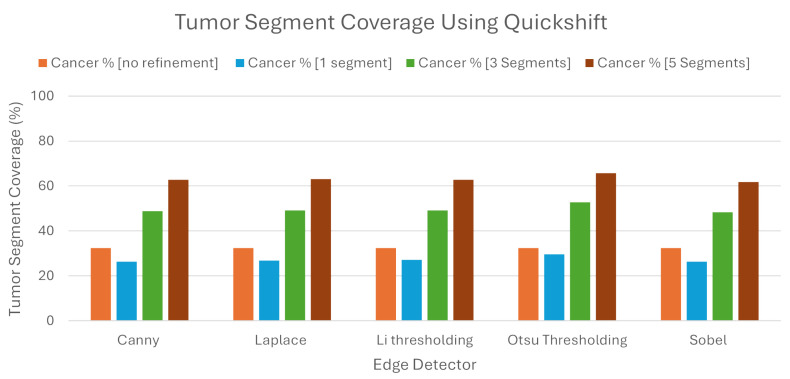
Tumor Segment Coverage average using Quickshift.

We can see from the Figure 10, that when we used Quickshift as the segmentation algorithm, the LIE produced explanations that had 32.41% TSC on average, without the proposed refinement. We must note here that the LIE took the three most important areas.

Following the introduction of the refinement mechanism, using again the three most important segments, a substantial improvement was observed, with the TSC average increasing to 49.68% across the five edge detection techniques. We must also note here that Otsu’s thresholding produced the best explanations compared to the rest of the edge detection algorithms, as the TSC with this technique averaged 52.74%, when the rest averaged below 49.2%.

To determine the best number of segments for generating meaningful explanations, we explored the impact of selecting the best single, three, and five segments using the refined LIE. Examining the Tumor Segment Coverage, we found that relying on a single segment yielded an average TSC of 27.26%, and employing five segments resulted in a TSC average of 63.22%. All of the results are presented in Table 2. As we can see, Otsu’s thresholding gave the best results in all of the experiments using Quickshift.

**Figure 11:**
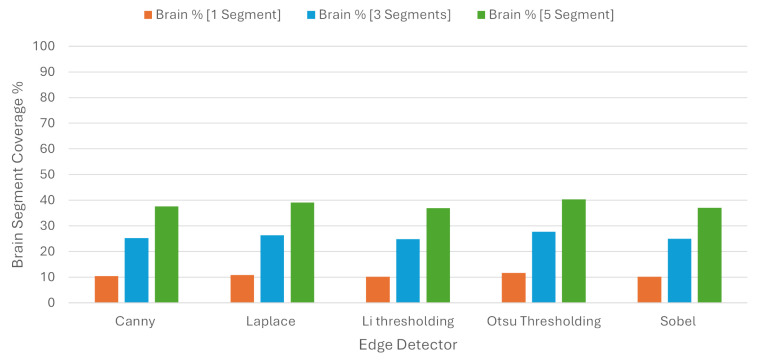
Brain Segment Coverage average using Quickshift.

In order to check the balance between coverage and specificity, we used the BSC, where one segment covered 10.71%, three segments covered 25.85%, and five segments covered 38.23% of the brain region on average. These results are presented in Table 3 and in Figure 11.

Regarding Felzenszwalb, our refinement seemed to work slightly better than the algorithm without it. To be more specific, when the LIE produced explanations, the average TSC was 36.43%. We must note here that this average was higher than the percentage found regarding Quickshift as the image segmenter. As noted before, the same average with Quickshift was 32.41%.

While this algorithm produced better explanations without the proposed refinement, when we tried to intervene, the new explanations produced had, once again, better TSC percentages, but the results were quite dissapointing. Our refinement showed roughly a 6% higher TSC, with the average being 42.64%.

**Figure 12:**
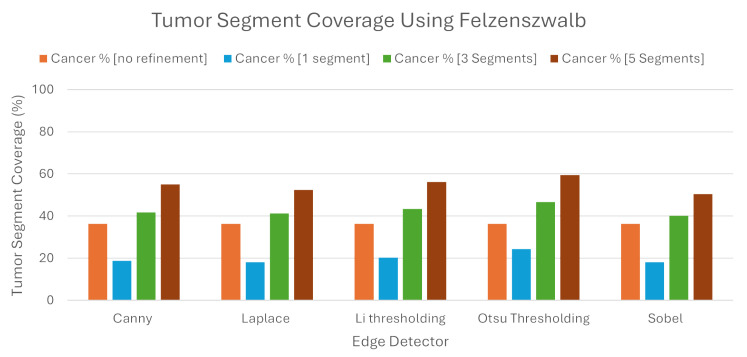
Tumor Segment Coverage average using Felzenszwalb.

As we can also see from the Table 4 and the Figure 12, the selection of one and five segments did not bring better results compared to the Quickshift experiment. Selecting the best segment had average TSC of 19.96%, while selecting the best five segments had only a 54.72% TSC. The same percentages in the previous experiment were approximately 10% higher in both selections. The one thing that must be noted here is that, once again, Otsu’s thresholding brought better results in the production of explanations compared to the other methods.

**Figure 13:**
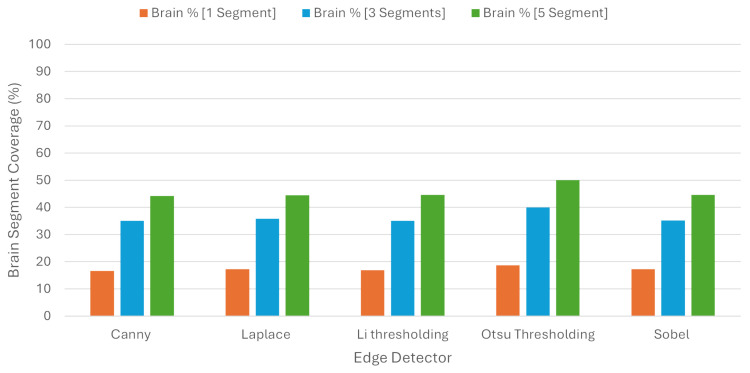
Brain Segment Coverage average using Felzenszwalb.

Consistent with the previous results, there was no improvement regarding the BSC. To be more explicit, for every experiment that we made regarding the number of segments, the percentage of the brain used was higher in all of them. As we can see from Table 5 and Figure 13, when we chose one, three, and five segments, the BSC percentages were 17.34 %, 36.23%, and 45.57%, respectively, while the same percentages using Quickshift were 10.71%, 25.85%, and 38.23%.

Not only werethe explanations produced less informative, but they used more brain area; so, in this case, this algorithm was not a better approach than Quickshift. The one thing that we should mention once more here is that, again, the proposed refinement produced better explanations than the original algorithm.

For the last experiment, Slic was used in order to separate the image into areas. This algorithm had the best results in all of the experiments and methods used. We must say here that we only compared this algorithm with Quickshift, having in mind that Felzenszwalb performed worse, so there was no need to compare it.

**Figure 14:**
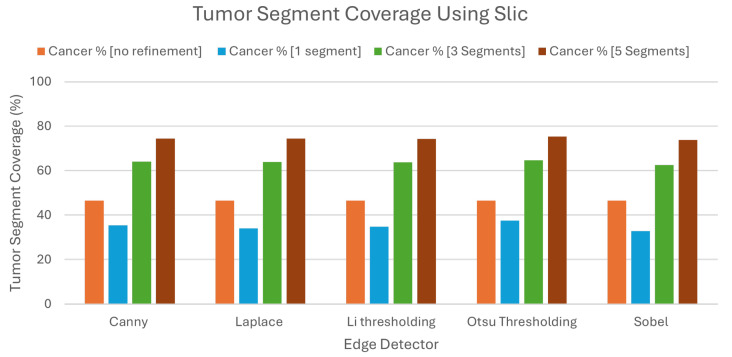
Tumor Segment Coverage Average using Slic.

We can clearly see in Table 6 and Figure 14 that all of the results were elevated. Without the proposed refinement, the LIE itself was capable of producing explanations that had a 46.53% TSC on average. This percentage was more than 10% higher than both of percentages Quickshift and Felzenszwalb.

With the refinement and the selection of the three most important segments, the TSC on the produced explanations rose to 63.77%. This percentage was even higher than the previous experiments when we picked the five most important segments. This means that this algorithm with the combination of our refinement was capable of producing better explanations even with a lower number of segments picked. Indeed, even when we picked only the best segment, the TSC percentage coverage was 34.9% on average, which is a value that is higher than the average TSC percentage when we used the Quickshift algorithm without the refinement and the best three segments (32.41%). The explanations produced with the selection of the five best segments had 74.42% TSC on average, leveraging the highest TSC on all of the experiments conducted.

**Figure 15:**
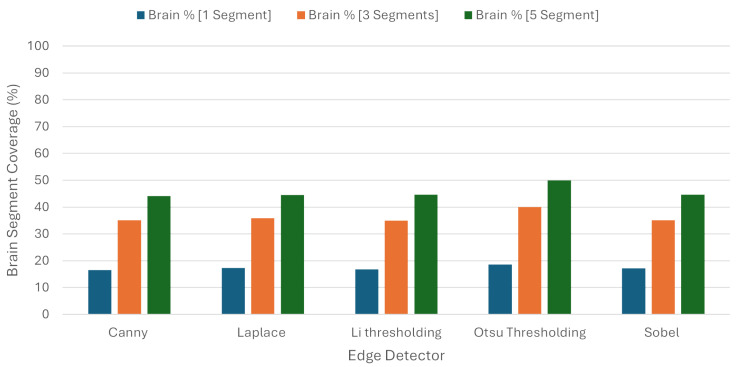
Brain Segment Coverage average using Slic.

Examining the BSC of the explantions, as shown in the Figure 15 and the Table 7, we can see that the percentages were slightly higher than Quickshift when selecting the best three and five segments. To be more precise, selecting three and five segments resulted in BSC average percentages of 27.7% and 44.67%, respectively, and in the same cases when using Quickshift, the same percentages were 25.85% and 38.23%.

In contradiction with previous statements, the selection of the best segment had a BSC of 10.3% on average, which is lower than the percentage found from every experiment conducted before in this study. This average, combined with the fact that the average TSC when selecting the best segment was 46.53%, assures that the refinement may produce better explanations than the original algorithm, even with the selection of less segments.

### 4.4. Statistical Evaluation

In this section, an investigation has been conducted to identify the existence of specific combinatory approaches that outperform others. In particular, we evaluated whether certain combinations of the LIE segmentation algorithms and brain area detectors would produce, statistically speaking, better results. As such, a thorough analysis on the performance of all of our segments/refinement combinations was performed. The performance criterion was considered as the accurate detection of tumor regions. Recall that a desirable outcome would be a high overlap of tumor areas with the *n* top-ranked areas according to the LIE outcomes.

**Figure 16:**
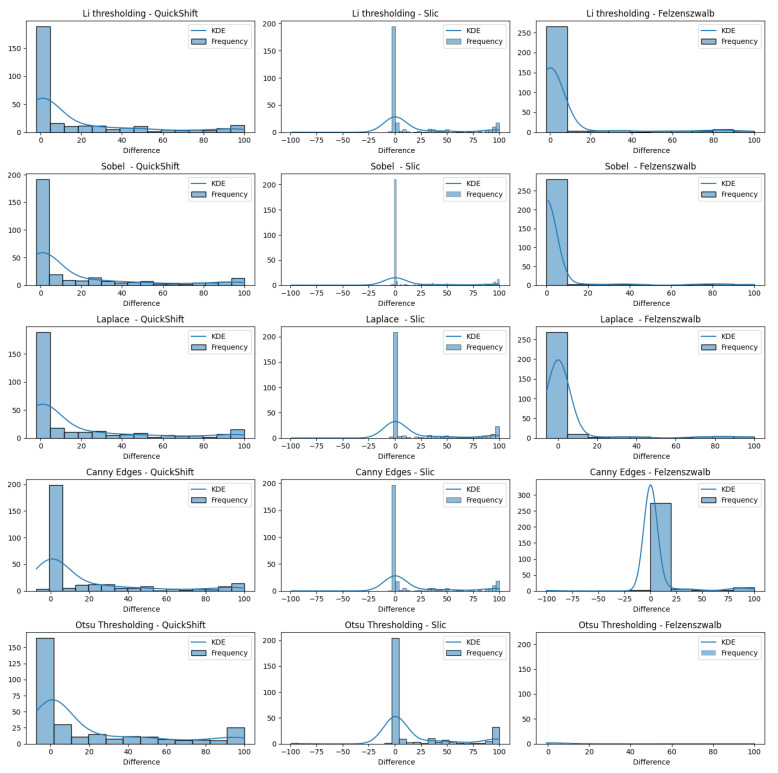
Improvements over the tumor segment coverage before and after the refinement process.

In Figure 16, we present varius histograms summarizing the difference in tumor segment coverage, for each image, before and after using our refinement method. A positive result indicates that the refinement approach resulted in a higher percentage of tumor-related areas to appear on the top-3 segments provided by the LIE. Each histogram represents a different combination of segment and brain region detector, providing a comprehensive view of the performance across all tested configurations.

**Figure 17:**
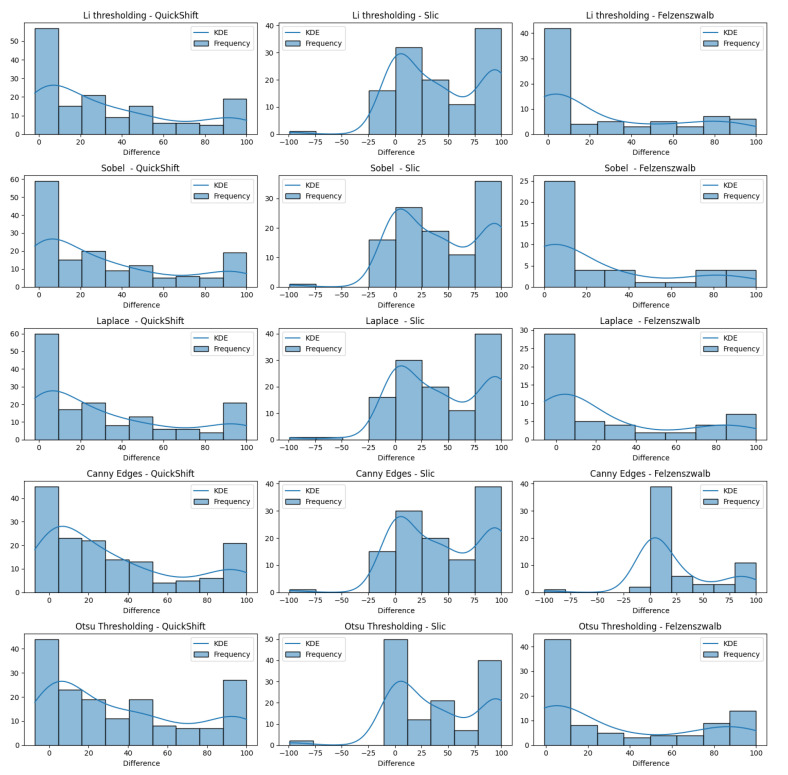
Improvements over the Tumor Segment Coverage before and after the refinement process for images with absolute difference values greater that 0.01.

It is important to note that a significant subset of images exhibited minimal variation in terms of the TSC, with changes of less than 1%. To better assess the effects of our refinement mechanism, we concentrated on the subset of images that demonstrated a more substantial variation in the TSC, exceeding 1%. Figure 17 demonstrates this case.

The results indicate that all of the edge detectors, in combination with the QuickShift segmentation algorithm, led to a positive improvement regarding the tumor area detection. A similar situation was observed when using Felzenszwalb as the segmenter, with the exception of the Canny edges detector for the refinement; less than 10 images obtained worse results. This is happening due to the weakness of the edge detector to create a brain mask that covers the brain area in some images (see Section 4.5). Something else that should be noticed is that, even when we saw a positive impact regarding the TSC difference, the improvement in the majority of the images ranged only from 0 to 10%.

Now, when we used Slic as the image segmenter, the histograms were quite different. We can see in each of the histograms regarding Slic that there was one or two images that had a negative impact that ranged from 50 to 100%, not being able to produce a proper explanation. The reason that this is happening, as mentioned before, is the inability of the edge detectors to produce a proper brain mask that covers the brain area. We can also see that there were about 15 images that had a negative impact ranging from 0.1 to 5%, but they were just being placed in the bin ranging from −25 to 0. Having this in mind, we can also see that the majority of the explanations had positive impacts, and most of them had an improvement in their TSCs ranging from 75 to 100%, excluding the case using Otsu’s thresholding as the edge detector.

The analysis reveals that all proposed combinations led to a general positive impact on tumor area detection. Also, our refinement approach enhanced the accuracy and reliability of identifying tumor regions, thereby validating the effectiveness of our method. When applying our refinement proccess, the regions of interest contained tumor areas in a higher percentage than before, enhancing the overall interpretability and usefulness of the model’s predictions in a clinical setting.

We also conducted a Kruskal–Wallis statistical test regarding the differences before and after the refinement method for each of the combinations used in order to check if there was a specific combination that would produce better results than the rest.

**Figure 18:**
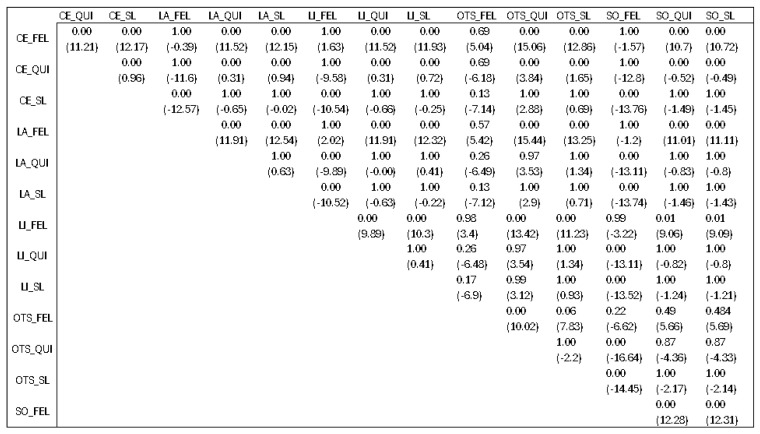
Statistical measurements between techniques, with respective *p*-values and mean differences.

The finding of this statistical analysis did not show us which combination is better than the rest, but which combinations should be avoided. Checking previous results presented in Section 4.3, we would say that the best algorithm is Slic when talking only about the segmenter. After looking at the results presented in Figure 18 with *p*-values and mean differences (denoted in the parenthesis) between different combinations, we can come to a conclusion.

The first characters before the underscore denote the edge detection techniques, Canny edges (CEs), Laplacian edges (LAs), Tthresholding LI (LI), Otsu’s thresholding (OTS), and Sobel (SO). The characters after the underscore denote the segmentation algorithm used from the LIE, Slic (SL), Quickshift (QUI), and Felzenszwalb (FEL).

There was not a specific technique that outperformed the rest, but the combinations that had Felzenszwalb as the image segmenter were worse than the others. This means that, statistically speaking, if we pick Slic or Quickshift as image segmenters, we would not have a big difference for the explanations produced. But, if we choose Felzenszwalb as the segmenter, the explanations that will be produced will not give us a clear understanding behind the model’s decision.

### 4.5. Advantages and Limitations

The obtained results, see Section 4.3, suggest that there is potential in refining LIMEs explanations using image processing approaches. In order to quantify the improvement in interpretability of the black box models, two performance metrics must be considered simultaneously: TSC and BSC. Both values are likely to increase if we maintain more segments for the analysis. Yet, presenting a large area of healthy brain tissue as an explanation for a positive tumor detection is counterintuitive. This scenario is further explained in the text bellow.

The Slic algorithm appears to be the most appropriate image segmentation technique. The average TSC score was higher than the rest of the image segmentors. As shown in Table 6 and Table 7, both the TSC and BSC scored higher when using five segments, with average scores of 74.42% and 44.67%, respectively. Despite the high TSC score, the BSC score of 44.67% advises against using five segments. Practically speaking, almost half of the brain tissue (BSC score of 44.67%) was presented as important for the decision, and approximately one-fourth of the tumor was not included in the suggested areas (1 − TSC = 25.58%).

Considering the above situation, utilizing the top-3 segments appears to be a better alternative. TheTSC and BSC scores were 63.77% and 27.7%, respectively. In other words, the areas presented for explanation cover one-forth of the brain tissue, and approximately one-third of the tumor is not included in the suggested areas (1 − TSC = 36.23%). As such, the use of three segments emerges as an appropriate choice, finding a balance between avoiding the overuse of non-informative brain regions and offering insightful explanations.

While the proposed refinement mechanism has shown improvement in explainability, certain limitations should be acknowledged. First of all, the accuracy and precision of the initial segmentation accomplished by the chosen algorithms directly affects how effective the refinement technique is. Any inaccuracies or inconsistencies in the segmentation output can propagate through the refinement process, compromising the quality of the explanation and possibly resulting in incorrect interpretations.

Factors influencing segmentation quality include the algorithm’s parameter settings, image characteristics (i.e., resolution, contrast), and the presence of noise or artifacts in the input images. In scenarios where the segmentation algorithms fail to accurately define tumor boundaries or distinguish between tumor and non-tumor regions, the subsequent refinement process may struggle to isolate relevant segments, resulting in explanations that are incomplete or erroneous.

**Figure 19:**
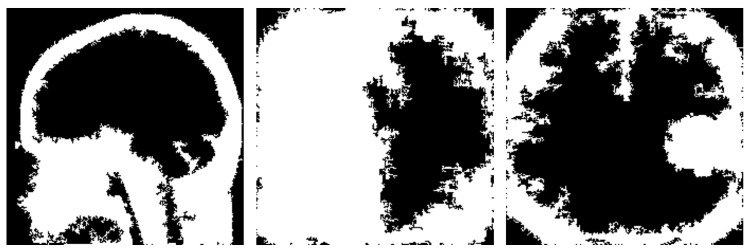
Instances of wrong brain mask production.

Another notable drawback observed in the proposed refinement mechanism is the potential inconsistency in creating the brain mask (see examples in Figure 19). The method relies on the edges detected by the different techniques and subsequently extracting the largest contour as the brain mask. However, in some instances, this process may yield inconsistent results.

One issue arises when the detected edges fail to accurately define the boundaries of the brain, resulting in incomplete or fragmented contours. Consequently, the extracted brain mask may cover only a portion of the actual brain region or extend beyond its boundaries, leading to false interpretations during explanation generation.

To address these challenges, future iterations of the refinement mechanism could explore alternative approaches for brain mask generation, such as incorporating machine learning-based segmentation methods or integrating feedback mechanisms to iteratively refine the mask based on user input. The utilization of multimodal imaging and omics data could further refine the explainability of these models by offering a more detailed and comprehensive understanding of tumor characteristics. This approach may also aid in the identification of biomarkers, enhancing the precision of brain tumor analysis. Additionally, robust preprocessing techniques and parameter tuning may help improve the reliability and consistency of edge detection algorithms, thereby enhancing the overall effectiveness of the refinement process.

## 5. Conclusions

Throughout this research, our primary objective was to enhance the interpretability of deep learning models in medical image analysis, particularly in the context of brain tumor detection. We aimed to address the challenge of understanding and explaining the predictions generated by some pretrained models, making an effort to bridge the gap between complex algorithmic outputs and human interpretability. Specifically, we sought to investigate the effectiveness of the LIE in providing explanations for model predictions and to propose a refinement mechanism to augment the specificity and accuracy of these explanations.

Through a series of experiments and analyses, we have demonstrated the efficacy of our proposed refinement mechanism in improving the interpretability of deep learning models for brain tumor detection. By integrating the LIE with segmentation algorithms and edge detection techniques, we achieved more precise and informative explanations for model predictions. Our results highlight the importance of refining the initial explanations provided by deep learning models, particularly in complex medical imaging tasks.

Although the results demonstrate the effectiveness of the refining mechanism, it is important to recognize the limits that have been noted, especially with regard to the consistency of brain mask production and image segmentation. To ensure reliable and effective brain mask production, further work should be focused on improving the refinement, possibly incorporating machine learning-based segmentation methods or integrating feedback mechanisms as stated before.

Overall, this work is a positive step toward improving the interpretability and transparency of deep learning models used in medical image analysis. The development of trustworthiness and the ease of integrating these models into clinical decision-making processes will depend on continuous efforts to improve explainability mechanisms as the field progresses.

## Figures and Tables

**Table 1:** Performance metrics for each pre-trained model after k-fold validation.

	Inception	ResNet50v2	NasNetLarge
Precision	0.9397	0.9826	0.9473
Accuracy	0.9597	0.9596	0.9711
Recall	0.9402	0.9664	0.9536
F1 Score	0.9496	0.971	0.9591

**Table 2:** Average metrics for Tumor Segment Coverage using Quickshift as image segmenter.

	Tumor Segment Coverage %			
**Edge Detector**	**No Refinement**	**1 Segments**	**3 Segments**	**5 Segments**
Canny	32.41	26.41	48.9	62.7
Laplace	32.41	26.9	49.21	63.11
Li	32.41	27.14	49.20	62.82
Otsu	32.41	29.58	52.74	65.72
Sobel	32.41	26.3	48.38	61.74
Average	32.41	27.26	49.68	63.22

**Table 3:** Average metrics for Brain Segment Coverage using Quickshift as image segmenter.

	Brain Segment Coverage %		
**Edge Detector**	**1 Segments**	**3 Segments**	**5 Segments**
Canny	10.44	25.33	37.62
Laplace	10.94	26.44	39.15
Li	10.25	24.81	36.92
Otsu	11.68	27.71	40.34
Sobel	10.22	24.97	37.13
Average	10.71	25.85	38.23

**Table 4:** Tumor Segment Coverage using Felzenszwalb as image segmenter.

	Tumor Segment Coverage %			
**Edge Detector**	**No Refinement**	**1 Segments**	**3 Segments**	**5 Segments**
Canny	36.43	18.75	41.7	55.08
Laplace	36.43	18.2	41.31	52.45
Li	36.43	20.32	43.33	56.17
Otsu	36.43	24.43	46.74	59.42
Sobel	36.43	18.1	40.11	50.51
Average	36.43	19.96	42.64	54.72

**Table 5:** Average metrics for Brain Segment Coverage using Felzenszwalb as image segmenter.

	Brain Segment Coverage %		
**Edge Detector**	**1 Segments**	**3 Segments**	**5 Segments**
Canny	16.6	35.09	44.18
Laplace	17.32	35.86	44.47
Li	16.87	35.04	44.63
Otsu	18.64	39.97	49.97
Sobel	17.25	35.18	44.61
Average	17.34	36.23	45.57

**Table 6:** Tumor Segment Coverage using Slic as image segmenter.

	Tumor Segment Coverage %			
**Edge Detector**	**No Refinement**	**1 Segments**	**3 Segments**	**5 Segments**
Canny	46.53	35.45	63.98	74.41
Laplace	46.53	33.96	63.96	74.34
Li	46.53	34.79	63.74	74.22
Otsu	46.53	37.54	64.67	75.37
Sobel	46.53	32.75	62.52	73.76
Average	46.53	34.9	63.77	74.42

**Table 7:** Average metrics for Brain Segment Coverage using Slic as image segmenter.

	Brain Segment Coverage %		
**Edge Detector**	**1 Segments**	**3 Segments**	**5 Segments**
Canny	9.83	26.83	43.63
Laplace	10.57	28.07	45.1
Li	9.35	25.93	42.83
Otsu	11.91	31.2	48.86
Sobel	9.86	26.46	42.91
Average	10.3	27.7	44.67

## Data Availability

The dataset utilized in this study is the “Brain Tumor Dataset” https://www.kaggle.com/datasets/preetviradiya/brian-tumor-dataset (accessed on 10 August 2023) obtained from Kaggle. This publicly available dataset consists of 4602 MRI images capturing random instances of the brain. The images are categorized based on the presence or absence of a brain tumor, offering a wide range of samples for testing and training. The dataset includes various perspectives like axial, coronal, and sagittal views. To maintain consistency, only JPEG images in grayscale format were retained for analysis, ensuring a standardized input for the models and enhancing the reliability of the study’s outcomes.

## Abbreviations

The following abbreviations are used in this manuscript:

AI	Artificial Intelligence
MRI	Magnetic Resonance Imaging
CNN	Convolutional Neural Network
CTG	Cardiotocographic
SVM	Support Vector Machine
XAI	Explainable Artificial Intelligence
LIME	Local Interpretable Model-Agnostic Explanations
ReLU	Rectified Linear Unit
ResNet	Residual Network
LIE	Lime Image Explainer
TP	True Positive
TN	True Negative
FP	False Positive
FN	False Negative
TSC	Tumor Segment Coverage
BSC	Brain Segment Coverage
